# Recollections of Thirty-Nine Years in the Army

**Published:** 1899-03

**Authors:** 


**Recollections of Thirty-nine Years in the Army.** By Sir CHARLES ALEXANDER GORDON, K.C.B. Pp. viii., 320. London: Swan Sonnenschein & Co., Limd. 1898.

This is a record of the services of a distinguished Army Medical Officer, extending uninterruptedly from the year 1841 to 1880. It is a plain statement of facts, without any adornment of style or any attempt at sensational writing. Surgeon-General Gordon has done long and faithful service to his country, and the five medals on his breast shown in his photograph in the frontispiece of his book attest that they were won in many an arduous campaign, and through many dangers from battle besides the risks run in some of the most deadly climates in which British soldiers are called upon to serve.

Sir Charles has purposely avoided all reference to medical or surgical matters in his narrative, although no man is more capable of dealing with them in a masterly manner.

A glance at the page of contents of the book will show the reader what stirring events he has witnessed. Beginning as Assistant-Surgeon of that gallant corps the 3rd Buffs, in 1841, he joined his regiment in India, serving in the Gwalior campaign in 1843. Returning home in 1845, he volunteered for service on the West Coast of Africa, which at that time held out to young medical officers the prospects of early promotion if they survived the risks of the climate. His service there resulted in some damage to his health; but he was back in India in 1852, but only for a short service, as he was compelled to return home again on sick leave. In the bracing air of his native land he soon recovered, and again went to India to find the great Sepoy Mutiny in full force in 1857. Through a great part of the outbreak he served principally with the Jounpore Field Force. He was present at the capture of Lucknow, and returned to England in 1859. While serving at home in 1860 he was suddenly ordered to embark for China, where he served in the stirring events at Tientsin and the Taku Forts, meeting, among others, with General Gordon, of Khartoum celebrity, then a Captain in the Royal Engineers. After a short service at home he returned to India for the fourth time.

In the Franco-German War of 1870 Gordon was appointed Medical Commissioner attached to the French Army, his duties being to report to the War Office on certain specified points relating to military organisation in the field. He remained in Paris during the whole period of the memorable siege, and to the end of the war. His descriptions of the scenes he witnessed are most thrilling.

Subsequently he was again ordered to the East. This time to Burma, and afterwards to the Madras Presidency of India.

It will be seen that in the long service of this most energetic army medical officer he enjoyed very little comparatively of the pleasure of home service, and it is gratifying to know that after so much exposure to the dangers of malaria and to many other risks he returned home in fairly good health. In 1880 his honourable service in the army came to an end, and having received the reward for distinguished military service, he retired as a Surgeon-Major-General; and in the Queen's Jubilee year he received from his sovereign the distinction of K.C.B., which he had so faithfully earned.

We heartily commend this book as very pleasant reading.

